# High Incidence of Cataracts in the Follow-Up of Patients Undergoing Percutaneous Coronary Intervention for Chronic Coronary Total Occlusion

**DOI:** 10.3390/jcm10215002

**Published:** 2021-10-27

**Authors:** Ricardo Rivera-López, Celia García-López, José M. Sánchez-Moreno, Rafael A. Rivera-López, Julio Almansa-López, Ricardo Rivera-Fernández, Eduardo Molina-Navarro, Miriam Jiménez-Fernández, Santiago Ortiz-Pérez, José A. Ramírez-Hernández

**Affiliations:** 1 Cardiology Department, Virgen de las Nieves University Hospital, 18014 Granada, Spain; rfriveralopez@gmail.com (R.R.-L.); josemanuel34sm@gmail.com (J.M.S.-M.); edumolina64@hotmail.com (E.M.-N.); mirijf84@hotmail.com (M.J.-F.); ramirezj@ugr.es (J.A.R.-H.); 2 Biosanitary Research Institute ibs.GRANADA, 18012 Granada, Spain; drsantiagoortiz@gmail.com; 3 Department of Ophtalmology, University Hospital Virgen de las Nieves, Av. de las Fuerzas Armadas, 2, 18014 Granada, Spain; 4 University of Granada (UGR), 18016 Granada, Spain; rafaelriveralopez@correo.ugr.es; 5 Radiophysics Department, Virgen de las Nieves University Hospital, 18014 Granada, Spain; jalmansa.lopez@gmail.com; 6 Intensive Care Department, University Hospital of Jaén, 23007 Jaén, Spain; rriverafernandez@gmail.com; 7 Medicine Department, University of Granada (UGR), 18016 Granada, Spain

**Keywords:** cataracts, ionizing radiation, chronic coronary total occlusion, percutaneous coronary intervention

## Abstract

Development of cataracts is a well-known adverse effect of ionizing radiation, but little information is available on their incidence in patients after other medical procedures, such as cardiac catheterizations. The study objective was to determine the incidence of cataracts in a cohort of patients undergoing percutaneous coronary intervention (PCI) for chronic coronary total occlusion (CTO) and its association with radiation dose. The study analyzed the incidence of cataracts during the follow-up of 126 patients who underwent chronic total coronary PCI, using Cox regression to identify predictive factors of cataract development. The study included 126 patients, 86.9% male, with a mean age of 60.5 years (range, 55.0–68.0 years). Twenty-three (18.2% *n* = 23) developed cataracts during a mean follow-up of 49.5 months (range 37.3–64.5 months). A higher incidence was observed in patients who received more than 5 Gy (29.0% vs. 14.7%, Hazard ratio (HR = 2.84 [1.19–6.77]). Multivariate analysis revealed a relationship between cataract development during the follow-up and a receipt of radiation dose >5 Gy (HR = 2.60, 95% confidence interval [CI 1.03–6.61]; *p* = 0.03), presence or history of predisposing eye disease (HR = 4.42, CI:1.57–12.40), diabetes (HR = 3.33 [1.22–9.24]), and older age, as in >57 (HR, 6.40 [1.81–22.61]). An elevated incidence of cataracts was observed in patients after PCI for CTO. The onset of cataracts is related to the radiation dose during catheterization, which is a potentially avoidable effect of which operators should be aware.

## 1. Introduction

Percutaneous coronary intervention (PCI) is a well-established treatment of acute and chronic ischemic heart disease. Increasing numbers of patients undergo this intervention [1], and technological improvements have increased success rates [2].

There has been a corresponding increase in the performance of PCI for chronic coronary total occlusion (CTO) [2] after the demonstration of its usefulness in selected patients [3,4,5]. This is a technically complex procedure that requires specific material, specially trained staff, a large amount of contrast, and long fluoroscopy times with a consequent increase in the radiation dose [6,7,8,9].

Adverse effects of ionizing radiation are well documented, including stochastic effects (e.g., the development of certain types of cancer) and deterministic effects (e.g., radio-induced cataracts, erythema, infertility, etc.) [10].

The development of cataracts due to ionizing radiation was first described in survivors of the atom bombs exploded over Hiroshima and Nagasaki [11]. There are increasing concerns about exposure to radiation at work and during medical examinations and procedures and about its possible mid- to long-term effects.

Various studies have shown that radiation doses previously considered safe can be responsible for the development of cataracts in humans [11,12,13,14,15]. Based on this evidence, the limit for occupational exposure to an equivalent dose to the crystalline lens has been reduced to 20 mSv/year [16].

Numerous authors have examined the frequency of cataract development in radiologists and interventional cardiologists, among other clinicians [13,14,15,16,17], but there have been few studies of patients undergoing procedures that involve ionizing radiation [18,19,20]. A search of the literature revealed no investigations that specifically studied patients after PCI. In this regard, CTO uses particularly high radiation doses [6,7,8,9], and patients may be especially susceptible to developing cataracts after this procedure.

The objectives of this study were to determine the incidence of cataracts after treatment by PCI for CTO, and to explore any relationship between radiation dose during catheterization and subsequent cataract development.

## 2. Materials and Methods

### 2.1. Study Population

This observational, retrospective cohort study included consecutive patients undergoing PCI for CTO at our center between 2013 and 2017. Exclusion criteria were: previous cataract diagnosis or follow-up <12 months (due to loss to follow-up or death), The study protocol was approved by the ethics committee of our center.

### 2.2. Study Variables

Data were gathered on: demographic characteristics; cardiovascular risk factors; radiation dose during PCI for CTO, reported as kerma-area product (P_KA_ [mGy·cm^2^]) and air kerma at the patient entrance reference point (K_a,r_ [mGy]); chronic corticoid consumption, i.e., uninterrupted intake for ≥3 months; cataract-related medical history, including uveitis, laser therapy in anterior segment or retina, receipt of intravitreal medication, eye surgery, eye disease, retinal detachment, diabetic retinopathy, and myopic chorioretinopathy.

### 2.3. Cataract Follow-Up and Identification

**Figure 1:**
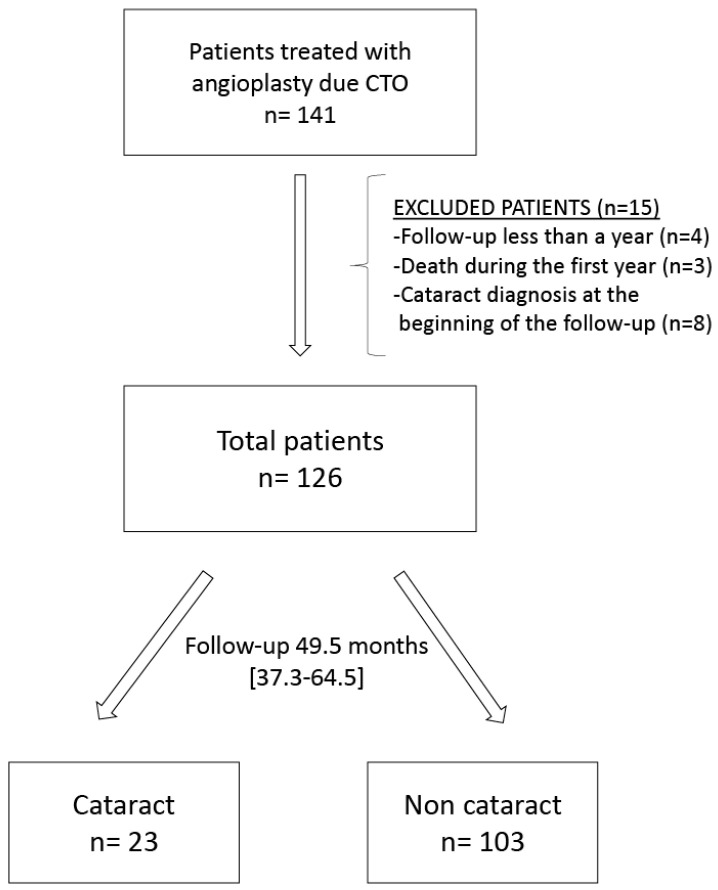
Flow chart of patient selection.

Patients underwent clinical follow up, recording referrals to an ophthalmologist for reduced visual acuity and defining the presence of cataracts by a reduction >0.5 in visual acuity and the biomicroscopic observation of crystalline lens opacification. Follow-up data were gathered by a cardiologist (RRL) and an ophthalmologist (CGL) from hospital clinical records, health center records, and telephone interviews with patients (Figure 1).

### 2.4. Dose Calculation

All catheterizations were performed in hemodynamic rooms with a Philips Allura Xper FD10 system (7.2.16, Philps Iberica, MARIA DE PORTUGAL, 1, Madrid, Spain). Dosimetry information for each procedure was collected from the data in the radiation dose structured reports (RDSR) of the equipment and was stored and processed using the OpenREM platform (https://openrem.org, last access 16 September 2021).

### 2.5. Statistical Analysis

Quantitative variables were expressed as medians (25–75 percentiles) and qualitative variables as absolute and relative frequencies. The Long-Rank test was used to compare cataract onset times over the follow-up, calculating the HR. For comparative analyses, quantitative variables were transformed into qualitative variables, grouping values by tertiles or quartiles. The fourth quartile of radiation dose was compared with the other three quartiles and the upper tertiles of age were grouped together to facilitate presentation of the data and to obtain a more homogeneous distribution.

Cox regression multivariate analysis was performed with cataract onset as the dependent variable, entering independent variables obtaining *p* < 0.20 in bivariate analysis and removing variables with *p* > 0.05.

## 3. Results

Figure 1 depicts the flow of patients through the study. The final study sample comprised 126 patients, 86.9% of whom males, with an age of 60.5 years (range, 55.0–68.0 years) and a median follow-up period of 49.5 [37.3–64.5] months.

### 3.1. Bivariate Analysis

The incidence of cataracts was 18.2% (*n* = 23). Patients developing cataracts were older, more often diabetic, more frequently had a predisposing eye disease, and received a higher radiation dose, with a marked difference between patients in the fourth quartile versus the other three quartiles (Table 1).

### 3.2. Multivariate Analysis

In the multivariate analysis (Table 2), cataract development was associated with older age, diabetes, predisposing eye disease, and the receipt of a radiation dose >5 Gy during catheterization. Smoking habit exited the model.

## 4. Discussion

In this study, around one-fifth of patients exposed to radiation during PCI developed cataracts within four years after the intervention. The risk of cataract onset was related to the radiation dose and was two-fold higher in patients in the upper dose quartile versus the other three quartiles.

CTO treatment is a reasonable option for selected patients and is associated with a high success rate [2,4,5]. Although complications associated with the radiation or contrast dose have previously been reported, the long-term effects of the radiation dose received by patients have been less well studied. These findings on the incidence of cataracts highlight the need to minimize the radiation dose selected for the procedure, inform the patients about the risks involved, and schedule regular ophthalmological examinations during the follow-up.

Data have been published on the incidence of cataracts among radiology and interventional cardiology personnel [12,13,14,15]. However, fewer studies have been performed on patients, focusing on those receiving direct cranial radiation and showing a high incidence of cataracts [17,18,19]. The effect of disperse radiation on the crystalline lens of patients is examined in the present study, with noteworthy results.

The present patient population had a high prevalence of risk factors for both ischemic heart disease and cataracts [21]. This suggests a multifactorial origin of cataracts, although the radiation dose has a highly significant effect when other variables are equal.

The radiation dose used in our series is elevated, as reported in other studies of CTOs [6,7,8,9]. Operators in these frequently long and complex procedures should be aware of the risks of radiation and must consider strategies to minimize the dose (e.g., low-dose mode, pulse or image rate reduction, use of collimation, etc.), implementing dose-monitoring programs and the new image-improvement technologies included in the most modern equipment [22,23,24,25].

Data on the prevalence and incidence of cataracts in our general population would be of interest but are difficult to gather, explaining the scant epidemiological publications on this issue [26]. The WHO estimated that 94 million people worldwide had visual disability due to cataracts in 2020 [27]. Various studies of Latino and Asian populations reported an increase in the prevalence of this disease with higher age, from 3.9% in the 55-to-64-year age group to around 92.6% over the age of 80 years [28,29,30]. However, most of these investigations were based on an active search for the presence of cataracts in the population, whereas our study only included patients seeking medical care for the loss of vision due to cataracts.

Ischemic heart disease and cataracts share type II DM, obesity and tobacco use as risk factors [28], and an association has been reported between the development of these two diseases [31]. This should be taken into account when interpreting our results, although the elevated incidence of cataracts in our population is striking, especially given the age of patients at its onset.

The prevention and early diagnosis of cataracts can be improved by determining risk factors in the general population and establishing whether therapeutic procedures using ionizing radiation contribute to increase its incidence and lower the age at onset. If this proves to be the case, preventive strategies could be adopted to protect the crystalline lens of patients from the radiation.

This study was limited to patients undergoing CTO treatment; however, account should be taken of the risk of cataracts in other procedures that involve similar radiation doses. Further studies with larger samples are warranted to verify the present findings.

### Limitations

Radiation doses were only studied during the CTO catheterization procedure, which is less precise than a full dosimetry history of the patient. However, this nondifferential classification bias was equally present in both groups. In addition, the Product kerma-area (P_KA_ [mGy·cm^2^) and air kerma at the patient entrance reference point (K_a,r_ [mGy]) were considered for the correlation between dose and cataract onset, and these magnitudes are not directly related to the dose in the crystalline lens, whose estimation is highly challenging. However, they are directly available from the equipment and easily measured for validation, and this is also a nondifferential bias. These magnitudes are also used to characterize interventional procedures and facilitate comparisons with other reports on CTO procedures. In addition, there was no untreated control group with similar characteristics for comparison of the incidence of cataracts over the follow-up period, which is an important limitation. Nevertheless, our observation of an incidence of 18% in a relatively young cohort is a noteworthy finding that warrants further research.

Finally, the sample size was relatively small, and the catheterization exposed all patients to elevated radiation doses. Hence, studies in larger patient samples with a wider range of radiation doses are required to verify these results and allow their extrapolation to different situations and settings.

## 5. Conclusions

The incidence of cataracts is elevated after PCI for CTO and is related to the radiation dose received during catheterization. Operators should be aware of this adverse effect and adopt measures to reduce the exposure of patients to radiation during this and similar procedures. Further studies in larger samples are needed to verify these results and explore measures for minimizing the radiation dose.

## Figures and Tables

**Table 1:** Baseline characteristics of the study population and their relationship with cataract development.

Variable	Total (*n* = 126)	Cataracts (*n* = 23)	*p*
Age			0.11
≤56 years	42	3 (7.1%)	
57–65 years	42	10 (23.8%)	
>65 years	42	10 (23.8%)	
Age > 56 years	84	20 (23.8%)	0.03
Sex female	14	3 (21.4%)	0.60
Hypertension	97	17 (17.5%)	0.58
Diabetes	50	15 (30%)	0.01
Dyslipidemia	78	13 (16.7%)	0.52
Chronic renal failure	8	1 (12.5%)	0.76
CABG	3	1 (33.3%)	0.37
Smoking habit	51	5 (9.8%)	0.08
Corticoid consumption	10	1 (10%)	0.52
Previous eye disease *	15	7 (46.6%)	0.01
Fluoroscopy time >40 min	67	14 (20.8%)	0.47
Radiation dose			0.11
≤1.9 Gy	31	6 (19.3%)	
1.95–2.60 Gy	32	5 (15.6%)	
2.7–4.9 Gy	32	3 (9.3%)	
≥5 Gy	31	9 (29.0%)	
Dose ≥ 5 Gy	31	9 (29.0%)	0.01

CABG: Coronary artery bypass grafting; *, e.g., uveitis, laser therapy in anterior segment or retina, intravitreal medication, or disease that reduces visual acuity such as retinal detachment, diabetic retinopathy, myopic chorioretinopathy, or eye surgery.

**Table 2:** Multivariate analysis, dependent variable “cataract onset”.

	Univariable Model		Multivariable Model	
	HR (95% CI)	*p* Value	HR (95% CI)	*p* Value
Dose > 5 Gy	2.84 [1.19–6.77]	0.018	2.60 [1.03–6.61]	0.044
Previous eye disease *	5.63 [2.26–14.04]	0.014	4.42 [1.57–12.40]	0.005
Age > 56 years	3.64 [1.08–12.29]	0.037	6.40 [1.81–22.61]	0.004
Diabetes	4.57 [1.81–11.56]	0.010	3.33 [1.22–9.24]	0.020
Smoking **	0.42 [0.16–1.12]	0.084	0.86 [0.29–2.59]	0.791

* as in Table 1. ** Variable that exited the model.

## Data Availability

The data analyzed in this study are available from the corresponding author upon reasonable request.
